# Supplementary material for: The Response Regulator OmpR Negatively Controls the Expression of Genes Implicated in Tilimycin and Tilivalline Cytotoxin Production in Klebsiella oxytoca
Source: Microorganisms. 2025 Jan 14;13(1):158. doi: 10.3390/microorganisms13010158 (PMC11767513; doi:10.3390/microorganisms13010158)
Supplement: Supplementary file 1 [file microorganisms-13-00158-s001.zip › microorganisms-3376019-supplementary.pdf]

Supplementary Table S1.

Primers used in this study

Primer	Sequence 5' → 3'	Target gene
For Gene deletion		
ompR-H1P1	GAACCTTTGGGAGTATAAACAATGCAAGAGAATTATAAGATTCTG **TGTAGGCTGGAGCTGCTTCG**	*ompR*
ompR-H2P2	GCGGCGAAAAGCGCACGCGTTTCATGCCTTAGAGCCGTCCGGGAC **CATATGAATATCCTCCTTAG**	
For Mutant Characterization		
ompR-MC-F	TGCTTTGTAACAATTTAGCCTGGA	*ompR*
ompR-MC-R	AAATAGGTCGTCACCAGGCTG	
For qPCR		
aroX-F	TGTTGCCTGCAAGATTGACG	*aroX*
aroX-R	ATGTGTGAACGGCCAAAACG	
npsA-F	AAATACGTGGCTTCCGCATC	*npsA*
npsA-R	TCCTGCGTGACATAACAAGC	
rrsH-F	CAGCCACACTGGAACTGAGA	*rrsH*
rrsH-R	GTTAGCCGGTGCTTCTTCTG	

The sequence related to the kanamycin cassette from the pKD4 template plasmid is emphasized in bold.
